# Temporal changes in the diazotrophic bacterial communities associated with Caribbean sponges *Ircinia stroblina* and *Mycale laxissima*

**DOI:** 10.3389/fmicb.2014.00561

**Published:** 2014-10-28

**Authors:** Fan Zhang, Jan Vicente, Russell T. Hill

**Affiliations:** Institute of Marine and Environmental Technology, University of Maryland Center for Environmental ScienceBaltimore, MD, USA

**Keywords:** diazotroph, diel cycle, symbiotic microbial community, Caribbean sponge, 454 pyrosequencing

## Abstract

Sponges that harbor microalgal or, cyanobacterial symbionts may benefit from photosynthetically derived carbohydrates, which are rich in carbon but devoid of nitrogen, and may therefore encounter nitrogen limitation. Diazotrophic communities associated with two Caribbean sponges, *Ircinia strobilina* and *Mycale laxissima* were studied in a time series during which three individuals of each sponge were collected in four time points (5:00 AM, 12:00 noon, 5:00 PM, 10:00 PM). *nifH* genes were successfully amplified from the corresponding gDNA and cDNA pools and sequenced by high throughput 454 amplicon sequencing. In both sponges, over half the *nifH* transcripts were classified as from cyanobacteria and the remainder from heterotrophic bacteria. We found various groups of bacteria actively expressing the *nifH* gene during the entire day-night cycle, an indication that the nitrogen fixation potential was fully exploited by different nitrogen fixing bacteria groups associated with their hosts. This study showed for the first time the dynamic changes in the activity of the diazotrophic bacterial communities in marine sponges. Our study expands understanding of the diazotrophic groups that contribute to the fixed nitrogen pool in the benthic community. Sponge bacterial community-associated diazotrophy may have an important impact on the nitrogen biogeochemical cycle in the coral reef ecosystem.

## Introduction

Coral reef ecosystems are well known for their high biodiversity and productivity, despite low ambient nutrient availability. Numerous studies on the nitrogen cycle in coral reefs have linked high local primary production to benthic biological activities, including the efficient recycling of nitrogen between algae and invertebrate hosts and benthic nitrogen fixing communities contributing to a “new” nitrogen source (Dugdale and Goering, 1967; Webb and Wiebe, 1978; O'Neil and Capone, 2009). Marine sponges are important habitat forming organisms in coral reef benthic communities. Their filter-feeding lifestyle can remove large amounts of organic particles in the size range between 0.2 and 10 μm, mainly bacterioplankton and phytoplankton, from the water column (Pile et al., 1996). These microorganisms are consumed as part of the sponge diet. Sponges also serve as hosts to many microbes that live inside the sponge mesohyl as their symbionts. The terms “symbiont” and “symbiosis” are used here consistent with Taylor (Taylor et al., 2007), according to the original definition by de Bary, to refer to two or more organisms found living together for a long period, and do not imply that the organisms benefit or harm each other. The density of microbial symbionts in sponges can reach a billion cells per ml volume, approximately three orders of magnitude higher than in the surrounding seawater (Taylor et al., 2007). Considering these high densities, symbionts are likely to play important roles in the hosts. Molecular tools and high throughput sequencing techniques have helped to overcome the constraints imposed by difficulty in culturing many of these symbionts, and have expanded our knowledge of the sponge microbiome, revealing their connections with host chemical defense, immunity and metabolism (Hentschel et al., 2012). Bacteria are the major driving force in the element biogeochemical cycle (Falkowski et al., 2008). In the sponge mesohyl, frequent water exchange between the sponge and outer environment can create an oxygen gradient and brings in a supply of nutrients (Hoffmann et al., 2005); these conditions may facilitate the essential redox reactions by symbiotic microorganisms (Fiore et al., 2010). The high abundance of microbial cells and suitable conditions are likely to result in significant nutrient flux mediated by the microbial community, which could be important for the local ecosystem.

A classic early study showed the transfer of a photosynthetic carbohydrate from symbiotic cyanobacteria to the sponge hosts (Wilkinson, 1983). The continuous influx of photosynthetic product that is rich in carbon but devoid of nitrogen could trigger the imbalance of C:N ratio in the symbiont-sponge system, leading to a nitrogen source deficiency. Field incubation experiment showed the uptake of ^15^N labeled ammonium and nitrate by both sponge cells and bacterial fractions and suggested the translocation of labeled nitrogen from bacteria to hosts (Fiore et al., 2013; Freeman et al., 2013). However, coral reef ecosystems are characterized by low dissolved nitrogen availability in the water column, conditions that might cause the sponge holobiont to seek an alternative nitrogen source to balance their budget. Nitrogen fixation, an anabolic pathway carried out only by prokaryotes, accounts for half of the reactive N supply that sustains ocean primary production (Gruber and Galloway, 2008). This pathway requires an anaerobic microenvironment and significant energy supply for N_2_ reduction. To provide suitable conditions for nitrogen fixation, some diazotrophs like *Anabaena* develop heterocysts as a spatial compartment to create the anaerobic condition; other groups like unicellular cyanobacterium *Cyanothece* conduct nitrogen fixation at night, temporally separated from the oxygenic photosynthesis that occurs during the daytime (Dixon and Kahn, 2004; Welsh et al., 2008). In a field study in 2007, we found consistently lower δ^15^N values from tissues of the sponge *Ircinia strobilina*, indicating that these sponge individuals obtain their nitrogen from nitrogen fixation, whereas samples from the sponge *Mycale laxissima* showed higher δ^15^N ratios, suggesting less reliance on nitrogen fixation. Subsequent molecular studies demonstrated the presence of diverse *nifH* genes from cyanobacteria along with heterotrophic bacteria in both sponges. However, the only *nifH* gene transcripts were those belonging to cyanobacteria (Mohamed et al., 2008). In the current study, we applied a high throughput sequencing method that allowed deeper coverage of the community, and expanded the sampling strategy to monitor the nitrogen fixing activities during a diel cycle. Through our study, we would like to provide more details to major questions regarding the diazotrophic communities associated with sponge hosts. Is the symbiotic community species specific? How stable is the community in the long term? Do active members shift over the diel cycle?

## Materials and methods

### Sampling collections

Tissue samples of *M. laxissima* and *I. strobilina* were collected by SCUBA diving at a depth of 20 m from Sweetings Cay, Bahamas (26° 33.78′N, 77° 52.89′W) in July 2012. Surface water temperature in the collection site was 26.7°C. Prior to collection, three large (1–5 kg) individuals of *M. laxissima* and *I. strobilina* were tagged for recurrent sampling. For each individual, 1 cm^3^ piece of tissue was collected with a sterile scalpel at local time 5:00 AM (dawn), 12:00 PM (noon), 5:00 PM (dusk), and 10:00 PM (night) for one diel cycle. To reduce the impact of tissue damage during sampling, small individual samples were taken from distant locations of the same sponge for each time point. During each night dive glow sticks were used instead of dive torches to prevent photosynthetic activity from interfering with nitrogen fixation. Samples for DNA and RNA extraction were preserved in RNAlater stabilization solution (Qiagen, Valencia, CA, USA) on board within 20 min after underwater collection prior to long-term storage at −80°C. Three seawater samples (5–10 L) from the sampling site were collected at noon in close proximity (1 m) to sampled sponges and filtered through 0.22 μm Sterivex filter units (Millipore, Billerica, MA, USA). Seawater samples were collected to compare the diversity of sponge nitrogen fixing bacteria with those found in the surrounding environment.

### Measurement of stable isotope composition

Sponge samples for δ^15^N measurement were collected from Sweetings Cay, Bahamas (26° 33.78′N, 77° 52.89′W) in July 2012 and from Conch Reef, Key Largo, Florida, USA, NE Caribbean (24° 57.11′N, 80° 27.57′W) in March 2010 and July 2011, prior to the collection of the sponge samples used in this study for the bacterial community analyses. Sponge samples for this purpose were drained and were rinsed three times with artificial seawater, then frozen at −20°C before processing. Three individuals of each sponge were lyophilized and grounded to fine powder. Samples (c. 1.0 mg) were packed in tin capsules for shipping and analyzed for nitrogen isotope ratios by continuous flow isotope ratio mass spectrometry at the UC Davis Stable Isotope Facility as described previously (Mohamed et al., 2008).

### Genomic DNA/RNA extraction and *nifH* gene PCR amplification

Total DNA and RNA from the three individuals of each sponge species collected during four time points from Sweetings Cay, Bahamas, July 2012 were extracted using a TissueLyser System (Qiagen), and an AllPrep DNA/RNA Mini Kit (Qiagen), combined with RNAase-free DNase treatment steps (Qiagen) for RNA samples following the manufacturer's protocol. Total DNA from seawater samples was extracted using a Power Water Sterivex DNA isolation kit (Mo Bio, Carlsbad, CA, USA) following the manufacturer's protocol. Nested PCR was used to amplify *nifH* gene fragments from genomic DNA (gDNA), and the cDNA derived from RNA as described below. For gDNA samples, *nifH* gene fragments were amplified by first round primers nifH32F (5′-TGAGACAGATAGCTATYTAYGGHAA-3′) and nifH623R (5′-GATGTTCGCGCGGCACGAADTRNATSA-3′) (Steward et al., 2004) at a concentration of 100 μM each because of the highly degenerate primers used for *nifH* genes covering 128 and 96 different combinations of nucleotide sequences. For RNA samples, the concentration of extracted RNA was measured using a Nanodrop spectrophotometer 2000 (Thermo Scientific, Waltham, MA, USA), and 100 ng of RNA template from each sample was added to RevertAid Reverse Transcriptase mix (Thermo Scientific) with primer nifH3 (5′-ATRTTRTTNGCNGCRTA-3′) as described previously by Zani et al. (2000). After reverse transcription, cDNA was amplified using first round PCR primers nifH3 and nifH4 (5′-TTYTAYGGNAARGGNGG-3′) at a concentration of 100 μM each. RNA samples without the RT step were included as PCR template to check for residual DNA in the RNA samples.

### Nested PCR amplification and amplicon sequencing

A total of 33 PCR product samples (three gDNA from filtered seawater, 24 cDNA samples from four time points of the six individuals and, six gDNA samples for each individual, pooled from four DNA extractions done at each of the four time points) from the first round were sent to Research and Testing Lab (Lubbock, TX, USA) and subject to a second round of PCR targeting a variable region (360 bp) encoding dinitrogenase reductase subunit using barcoded primer sets nifH1 (5′-TGYGAYCCNAARGCNGA-3) and nifH2 (5′-ADNGCCATCATYTCNCC-3′) (Zehr and Mcreynolds, 1989). Subsequent amplicon pyro-sequencing by 454 Life Science GS FLX + platform (Roche Diagnostics, Branford, CT, USA) generated about 3000 raw sequencing reads from each tagged sample.

### Sequence analysis pipeline

Initial data were processed using the mothur software package, following the guidelines and recommendations in the mothur manual (www.mothur.org) (Schloss et al., 2009) for sequence quality trimming, chimeric checking and denoising to generate a single fasta file. Sequence reads less than 300 bp, plus barcoding tag and primer information were subsequently removed using the “trim.seqs” command. The cleaned sequences were pre-clustered using Simultaneous Alignment and Tree Estimation using default setting for nucleotide analysis (Liu et al., 2009). Representative sequences from each cluster were blasted against the GenBank database using the blastn function to confirm sequence identity and non-*nifH* gene sequences were removed. In some of our cDNA samples, non-*nifH* gene sequences accounted for up to half of the total reads, and were classified as either 16S rRNA sequences from bacteria or 23S rRNA sequences from sponges. A possible explanation for this is that the RNA extraction included a large quantity of ribosomal RNA from microbial symbionts and the hosts, therefore the nested PCR used in our study could lead to reverse transcription and amplification of unintended rRNA sequences. After all corrections, we obtained 67,212 *nifH* sequences in 33 samples. Unique *nifH* sequences were translated into amino acid sequences (120 bp) using MEGA, then aligned to the reference *nifH* database from Marine Microbiology, University of California Santa Cruz (http://pmc.ucsc.edu/~wwwzehr/research/database/), built into a phylip-formatted distance matrix and clustered into OTUs at the 90% similarity level in translated amino acid sequences with the nearest neighbor method. Representative *nifH* sequences from each OTU were deposited in the NCBI database under accession numbers KM083066–KM083092 and raw amplicon sequence data were deposited in the NCBI-SRA database under BioSample accession SAMN02869232. 24 cDNA samples were sub-sampled according to the sample with the minimum number of reads to enable diversity comparisons among individuals and time points.

### Phylogenetic analysis of *nifH* genes

Translated amino acid sequences from representative OTUs and their top blast hits (GenBank database) were imported into ARB (Ludwig et al., 2004) for *nifH* gene phylogenetic analysis. Multiple sequence alignments were visually checked and improved manually using the ARB editor. The aligned *nifH* sequences (120 bp) were imported into PhyML 3.1 software package to construct a tree based on Maximum Likelihood method (Guindon and Gascuel, 2003). The robustness of the resulting tree topologies was evaluated by 1000 bootstrap replicates.

### Statistical analysis

Diversity metrics (observed OTUs, coverage, Chao1 estimator, Shannon index and, Simpson's inverse) were calculated for sequence data from sponge species and seawater. Diazotrophic communities in sponges were compared by nonmetric multidimensional scaling (nMDS). All analyses were performed using the mothur software package (Schloss et al., 2009). One-Way and multiple factorial ANOVA was performed in the Statistica 7.0 (StatSoft, Tulsa, OK, USA).

## Results

**Figure 1:**
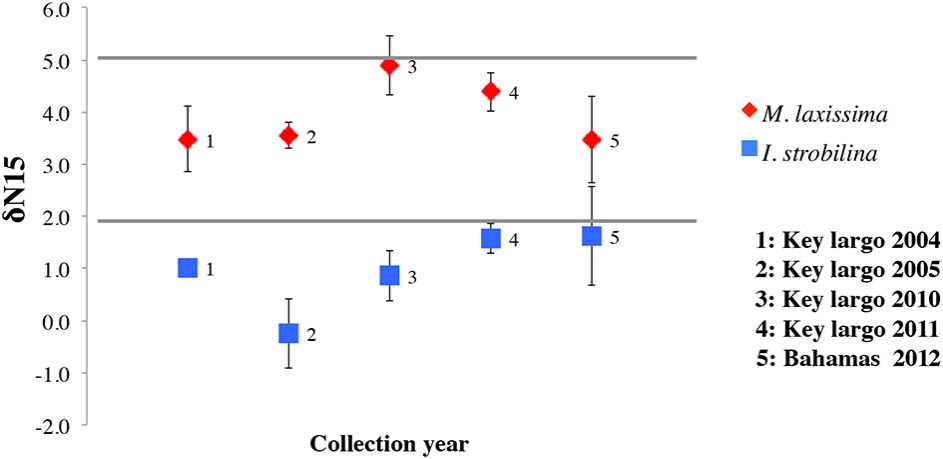
**δ15N values of sponges: *I. strobilina* (IS, square in blue), *M. laxissima* (ML, diamond in red).** Each point represents the mean δ15N value taken from three sponge individuals of the same species during different years. Error bars indicate the standard deviation of each mean calculation.

δ^15^N values from *I. strobilina* samples were 1.01 ± 0.97 (*SD*, *n* = 24), consistently lower than the values 3.86 ± 0.92 (*SD*, *n* = 24) from *M. laxissima* (ANOVA between sponge species, *p* < 0.01) and no significant difference (Multiple factorial ANOVA, *p* > 0.05) between the collection years (Figure 1). We also measured the δ^15^N data during the diel cycle. The outcome confirmed the difference in species level, but did not reveal any patterns over a 24 h time span (data not shown).

**Figure 2:**
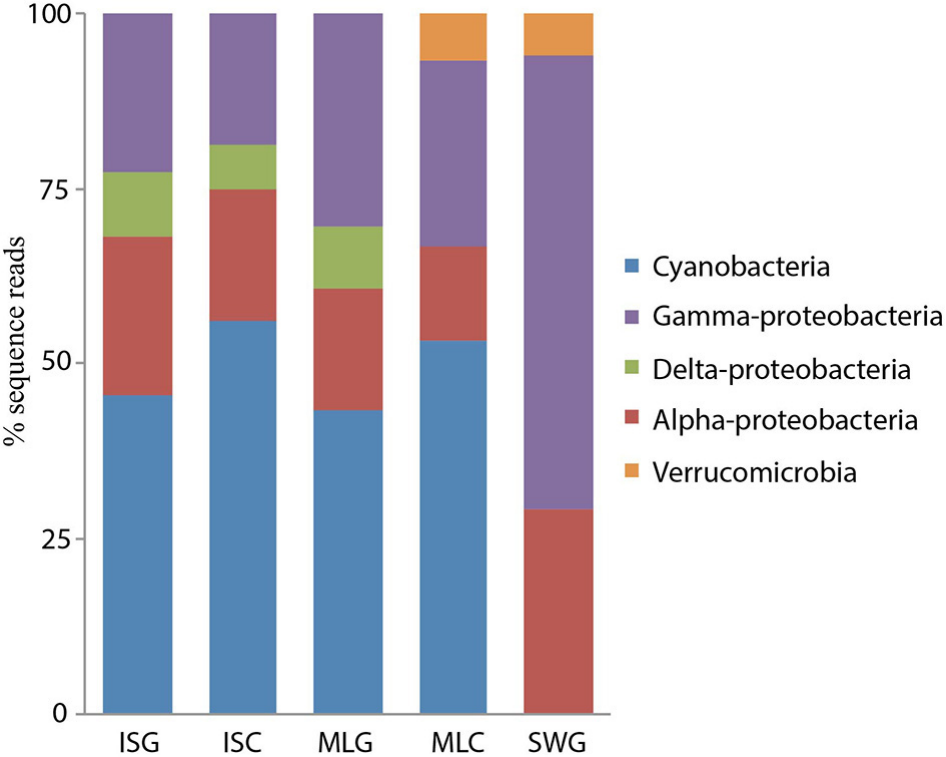
**Community structure *I. strobilina*, *M. laxissima* and seawater based on 90% translated amino acid sequences similarity of *nifH* genes from gDNA and cDNA sources.** (ISG: gDNA from *I. strobilina*, ISC: cDNA from *I. strobilina*, MLG: gDNA from *M. laxissima*, MLC: cDNA from *M. laxissima*, SWG: gDNA from seawater).

The diversity of the bacterial diazotrophic communities revealed by *nifH* gene sequences amplified from total DNA was quite similar between the two sponge species. In the current study, deep sequencing of *nifH* amplicons revealed a greater diversity of nitrogen fixing groups in the communities. Based on 90% amino acid sequence similarity, we recovered 22 OTUs from the sponge *I. strobilina*, and a slightly higher diversity (24 OTUs) from *M. laxissima* (Table 1). The communities were dominated by cyanobacteria, alpha-proteobacteria and, gamma-proteobacteria. Strict anaerobes belonging to delta-proteobacteria were also found. The diversity of *nifH* genes in the surrounding seawater was lower than in the sponges with 17 OTUs, all from heterotrophic bacteria (Figure 2) and, OTU composition was different from those detected in sponge samples (ANOVA, between sponges and seawater *P* < 0.001). Community compositions based on sequence reads from individuals were consistent in gDNA source, with no significant difference in both individuals (ANOVA between individuals, *p* > 0.05) and, species level (ANOVA between sponge species, *p* > 0.05). Detailed individual community compositions are shown in supplemental Figure S1.

**Table 1:** **Richness and dominance metrics for diazotrophic communities in sponges and seawater based on *nifH* gene sequences (OTU = 90% amino acid sequence similarity)**.

**Source**	**Observed OTUs (Sobs)**	**Number of reads**	**Expected OTUs (Chao1)**	**Simpson Inverse Index**	**Shannon Index**
***I. STROBILINA***					
gDNA	22	8290	26 (17–33)	12.2	3.2
cDNA	16	17855	18 (12–20)	6.9	2.6
***M. LAXISSIMA***					
gDNA	23	7383	24 (16–33)	12.5	3.2
cDNA	14	18537	17 (11–22)	6.1	2.5
**SEAWATER**					
gDNA	17	5147	21 (17–30)	9.2	2.9

The dominant cyanobacterial OTUs were found in all sponges, regardless of location and time of collection. In fact, the representative sequence from OTU1, belonging to the cyanobacterial genus *Leptolyngbya*, shared 100% identity with the DNA sequences found in the same sponge species collected in 2004 and 2005 at Conch Reef, Key Largo.

In the cDNA dataset, transcripts from cyanobacteria, alpha-proteobacteria and gamma-proteobacteria accounted for at least 80% of sequence reads in each sample. The diversity of *nifH* genes from the cDNA libraries was lower than from the gDNA libraries. 13 OTUs were shared between sponge species, although two gamma-proteobacterial OTUs were found exclusively in *M. laxissima* cDNA samples, and one cyanobacterial and one gamma-proteobacterial OTU were found only in *I. strobilina* samples. Cyanobacterial transcripts were found from filamentous cyanobacteria, including heterocyst-forming genera like *Anabaena*, and non-heterocyst forming genera like *Leptolyngbya* and, from unicellular cyanobacteria, closely related to *Cyanothece*. However, no group showed a consistent expression pattern corresponding to particular times in the light/dark cycle. Most transcripts from heterotrophic bacteria were classified either as alpha-proteobacteria closely related to aerobic genus *Xanthobacter* or as gamma-proteobacteria closely related to facultative anaerobe *Klebsiella* (Table 2). A complete list of OTUs found in this study is provided (Table S1) and their phylogenetic relationships with cultured nitrogen fixer and closest environmental clones are listed (Figure S2).

**Table 2:** ***nifH* gene OTUs found in high abundance in *I. strobilina* and *M. laxissima* sponge samples and their closest BLAST sequence matches**.

**Sponge- derived 90%-OTUs**	**No. of reads in each OTU per source**				**Closest BLAST match (accession no., % identity, source)**	**Closest cultivated microorganism (accession no., % identity, source)**
	**ISG**	**ISC**	**MLG**	**MLC**		
**OTU01**	673	3580	1310	4320	EU594242.1 (100%) Sponge RTMLH02	KC256775.1 (88%) *Leptolyngbya minuta*
**OTU02**	173	1967	989	2315	HM601491.1 (92%) Florida key reef water	AB264111.1(84%) *Cyanothece sp.*
**OTU03**	338	2215	282	627	EU594072.1 (96%) Sponge IS15S	HQ906641.1 (99%) *Mastigocladus testaurum*
**OTU04**	122	855	1688	3152	KF657100.1 (88%) Coral clone	FR669148.1 (84%) *Klebsiella sp.*
**OTU05**	428	558	155	663	GU594006.1 (95%) Freshwater lake	DQ439648.1 (95%) *Anabaena sphaerica*
**OTU06**	305	1458	198	1130	EU594012.1 (93%) Sponge IS3H07	CP000781.1 (98%) *Xanthobacter autotrophicus*

*ISG, gDNA from I. strobilina; ISC, cDNA from I. strobilina; MLG, gDNA from M. laxissima; MLC, cDNA from M. laxissima*.

**Figure 3:**
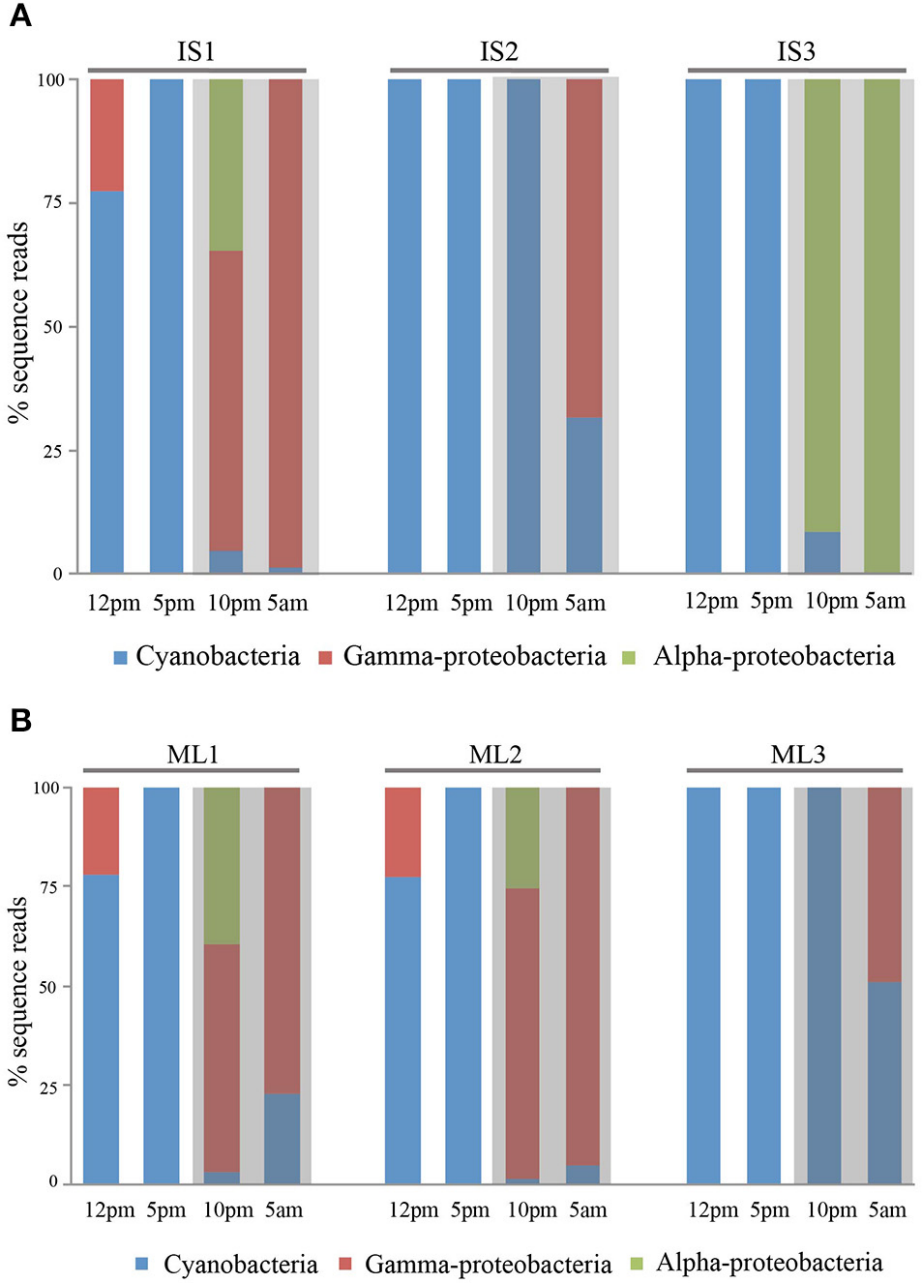
**(A)** Relative abundance of transcript reads from three individuals of *I. strobilina* in a diel cycle. IS1, IS2, IS3 represent three individual sponges. **(B)** Relative abundance of transcript reads from three individuals of *M. laxissima* in a diel cycle. ML1, ML2, ML3 represent three individual sponges.

We selected sequences from three major groups: cyanobacteria, gamma-proteobacteria and, alpha-proteobacteria. For these groups, we normalized sequence reads by minimum sample reads across all samples and compared the composition of the actively transcribed components of the community during day and night. When we combined transcript reads from six individuals of the two sponge species we found that cyanobacterial transcripts were dominant in the daytime, accounting for 94.1 ± 10.7% (*SD*, *n* = 6) of the total sequence reads from both sponge species. The percentage pattern changed significantly at night (One-Way ANOVA, *p* < 0.01), with 72.8 ± 37.4% (*SD*, *n* = 6) of transcripts deriving from heterotrophic bacteria (Figures 3A,B). In the species level, day/night difference was more significant for *I. strobilina* (ANOVA between symbiont species during day/night, *p* = 0.04) and less significant for *M. laxissma* (ANOVA between symbiont species during day/night, *p* = 0.11), largely due to a relative high proportion of cyanobacterial transcripts found in the nighttime sample of the third *M. laxissma* individual.

Within sponge samples, cyanobacteria transcripts were consistently dominant at dusk and heterotrophic bacteria were more abundant at dawn. In contrast, community structure at noon and, at night showed variation among different individuals at each time point. For example, two *I. strobilina* individuals showed dominance of heterotrophic bacterial transcripts at 10:00 PM, whereas individual 2 at the same time point had transcripts exclusively from cyanobacteria (Figure 3A).

**Figure 4:**
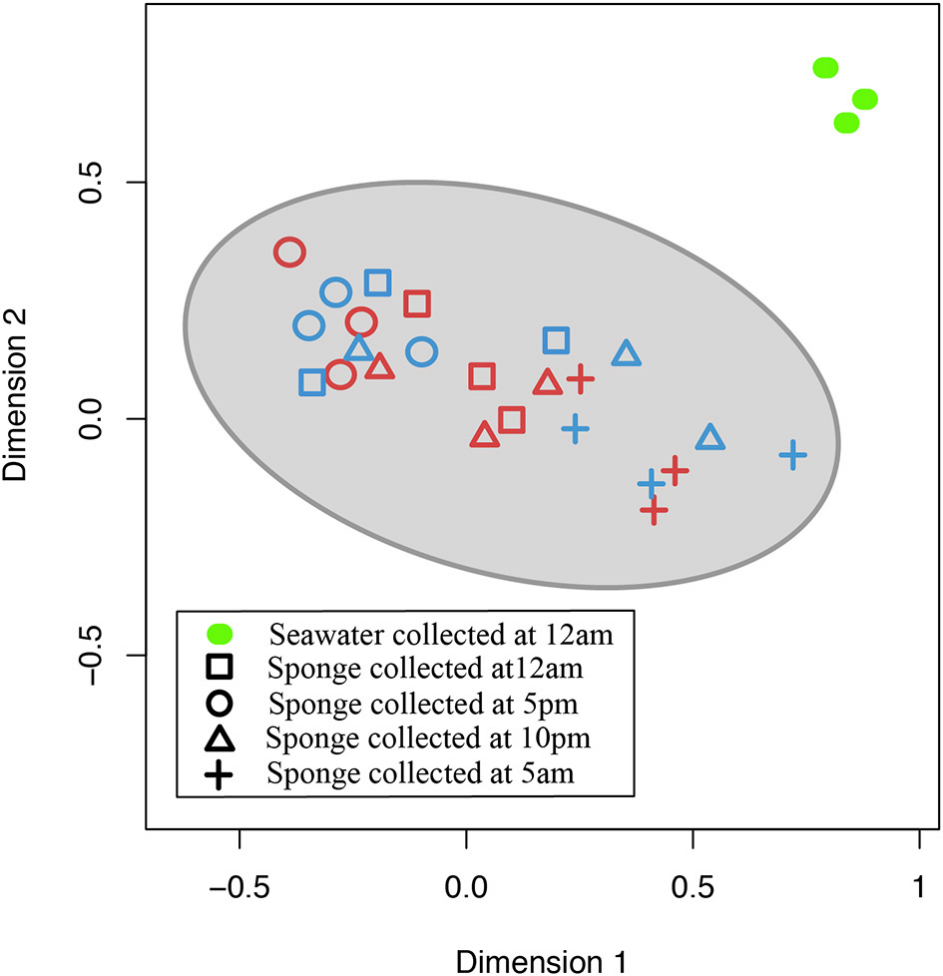
**Non-metric multidimensional scaling (nMDS) plot based on Bray-Curtis distances between cDNA samples from 4 different time points: 12 noon (squares), 5:00 p.m. (circles), 10:00 p.m. (triangles), 5:00 a.m. (crosses).** Blue color symbols represent samples from *I. strobilina*, red color symbols represent samples from *M. laxissima* and, green color solid oval symbols represent gDNA samples from surrounding seawater (Stress value = 0.10, R^2^ = 0.86).

In the nMDS plot of community structure, *nifH* gene sequences derived from sponge samples clustered together with no obvious distinction in sequences between the two sponge species, whereas *nifH* gene sequences derived from seawater samples were clearly separated from the sponge-derived sequences (Figure 4).

## Discussion

The δ^15^N values in the two sponges *M. laxissima* and *I. strobilina* were consistent over two sampling periods 8 years apart, suggesting that the sources of fixed nitrogen for each of these sponge species remain the same over long periods. The fact that *I. strobilina* is considered to be a high microbial abundance (HMA) sponge and *M. laxissima* to be a low microbial abundance (LMA) sponge (Reiswig, 1973, 1974) might translate to a relative higher prokaryote activity like nitrogen fixation in the *I. strobilina*. Based on field observation, adult *I. strobilina* individuals are generally denser than *M. laxissima* and show lower pumping rate, and this could create a less efficient oxygen-penetrated mesohyl for *I. strobilina* thus provide more anaerobic niches for nitrogen fixation by non-heterocyst cyanobacteria and heterotrophic bacteria. The δ^15^N values likely reflected the combined effect of biologically available fixed nitrogen input and loss in the holobiont. Studies on nutrient flux through sponges showed that sponges serve as a net source of nitrate (Jiménez and Ribes, 2007; Hoffmann et al., 2009; Fiore et al., 2013), although not much nitrate and ammonium release were reported for *M. laxissima* and *I. strobilina* (Southwell et al., 2008). This observation is potentially contradictory to the hypothesis that there is nitrogen deficiency in the sponge microbiome community that requires the activity of nitrogen fixing bacteria to provide additional fixed nitrogen. However, the net export of nitrate does not reflect the spatial heterogeneity in the sponge mesohyl (Webster et al., 2001; Radax et al., 2012). Whether the total sponge mesohyl is a nitrogen limited environment or not, carbohydrate input from photosynthesis and inorganic nitrogen species removal by ammonium oxidation and denitrification (Mohamed et al., 2009) likely requires localized replenishment of fixed nitrogen to the bacterial community. Previous research found similar low δ^15^N values in spongin fractions derived from sponge tissue, indicated the transfer of nitrogen to the hosts (Weisz, 2006). Measurement of δ^15^N values of bacteria and sponge cells separated from the same sample could provide more direct evidences on whether the hosts benefit more from fixed nitrogen produced by microbial symbionts.

In agreement with our previous study (Mohamed et al., 2009), we found similar nitrogen fixing communities in the two distantly related sponge species, suggesting that the difference in δ^15^N values observed in these two species might reflect differences in the rates of nitrogen-fixation rather than being the consequence of different nitrogen-fixing communities. In order to confirm the relatively lower δ^15^N data observed in *I. strobilina*, quantitation of *nifH* transcripts using real-time PCR could provide insights into the relative nitrogen fixation activities of the two similar nitrogen-fixing communities found in the two sponge species.

Studies of coral reef ecosystems have shown that nitrogen-fixing bacteria are widely distributed in the water column and corals (Hewson et al., 2007; Lema et al., 2014). Our results show a consistent presence of nitrogen-fixing cyanobacterial groups in marine sponges from two geographic locations of the Caribbean coral reef (Sweetings Cay, Bahamas and Key Largo, Florida). The fact that cyanobacterial-derived *nifH* transcripts are dominant in sponge samples collected during the daytime, suggests that cyanobacteria are key nitrogen-fixing symbionts in the two sponges. The coexistence of heterocyst-forming filamentous cyanobacteria and unicellular diazotrophic cyanobacteria in both sponges also suggests that, in order to protect nitrogenase from an oxidative environment, both spatial and, temporal separation strategies might be adopted by the community. However, when comparing the presence of heterocyst-forming filamentous and, unicellular cyanobacterial transcripts under light/dark conditions, neither group showed a consistent diel pattern in this study.

A previous study on *nifH* gene diversity associated with corals showed a high proportion of heterotrophic bacteria in those communities (Lema et al., 2014). In our study, high throughput sequencing revealed rare phyla like delta-proteobacteria that were not detected by our previous study in which we used a cloning and sequencing method. Unlike the cyanobacteria, nitrogen fixing heterotrophic bacteria seems to be more active in nitrogen fixation at night. The communities were not well conserved between the two sponge species or between sponges of the same species collected at each location, suggesting that the heterotrophic diazotrophic bacteria may not be as closely associated with their host as the cyanobacterial groups. The fact that, based on phylogeny (Figure S2), the closest environmental clones of many heterotrophic bacterial OTUs found in this study are connected with benthic community (i.e., associated either with coral, sea-grass or from the marine sediment) suggests that the heterotrophic nitrogen-fixing bacterial selection may be controlled by a “first come first served” process (Fan et al., 2012), which proposes that local geographic factors matter the most in shaping some bacterial communities.

A recent study on natural community in the Hawaii Ocean Time-Series station found a diel expression pattern in which photosynthesis related transcripts from *Prochlorococcus* peaked at dawn or dusk (Ottesen et al., 2014). Although not directly influenced by sunlight, nitrogen fixation is regulated by local environmental factors include pH, O_2_, NH^+^_4_ and, organic carbon availability which is strongly impacted by a photosynthetic process (Stacey et al., 1992). A study of diazotrophs in the open ocean found temporal patterns of *nifH* transcript abundance in different cyanobacteria phylotypes but no obvious pattern for heterotrophic gamma-proteobacteria (Church et al., 2005). Though the existence of horizontal transfer in *nifH* gene in proteobacteria (Cantera et al., 2004) could potentially complicate the phylogenetic assignment, so far no HGT of *nifH* gene between cyanobacteria and heterotrophs has been reported, thus the multiple taxon *nifH* gene expression in our study likely reflected bacterial responses to sporadic and transient environmental cues inside the hosts. Sponge mesohyl can undergo spatial gradients that fluctuate through active pumping (Hoffmann et al., 2005). The associated diazotrophic community must control N_2_ fixation under these oscillating environmental conditions. We detected diurnal patterns in those members of the bacterial community that are actively expressing nitrogen fixation genes. We speculate that this pattern may reflect a combined effect of energy supply from photosynthesis and oxic states in localized regions of the holobiont during the light/dark cycle. The low light intensity at dusk, at the end of the light cycle, may be a time at which energy is still available from the day-time photosynthesis to power nitrogen fixation in cyanobacteria while accumulated oxygen could limit nitrogen fixation in heterotrophic bacteria. Conversely, at dawn, at the end of the dark cycle, the energy gained from photosynthesis by autotrophs may be exhausted, and, bacterial respiration may create an anoxic state by oxygen consumption, favoring nitrogen fixation from heterotrophic bacteria. The other two time points in our dataset may reflect the intermediate state between the two scenarios described above, thus resulting in individual variation in active nitrogen fixers. Meanwhile, some sampling constraints might limit the interpretation of the current results. First, the stress effects incurred by each time of tissue collection on the sponge hosts and associated microbial communities are worth considering. Though the actual stress impacts are difficult to assess, an additional sampling point during the daytime showing the dominance of cyanobacterial *nifH* transcripts immediately after the dark cycle, would strengthen our hypotheses. Alternatively, the detection of the expression of stress gene marker like *hsp70* gene for the host (Lopez-Legentil et al., 2008) or *dnaK* gene (Glatz et al., 1999) for symbiotic cyanobacteria over the time course in future studies might provide insights into stress effects in the sponge during the course of sampling.

The imbalance of the global nitrogen budget indicates a potential underestimation of biological nitrogen fixation (Karl et al., 2002). Nitrogen fixation by non-cyanobacterial groups has been overlooked and could play an important role in marine environments (Moisander et al., 2014). Nitrogen fixation by non-cyanobacterial groups in sponges may also contribute to nitrogen input from the benthic community to nutrient limited coral reef ecosystems.

Our study showed that cyanobacteria are a dominant and consistent group in the diazotrophic community within sponges, and, various heterotrophic bacteria groups can be important components in the community. Composed of “core” cyanobacteria and flexible heterotrophic bacteria, nitrogen fixers in sponges represent an optimal combination to replenish the nitrogen pool.

### Conflict of interest statement

The authors declare that the research was conducted in the absence of any commercial or financial relationships that could be construed as a potential conflict of interest.
