# Supplementary material for: Temporal changes in the diazotrophic bacterial communities associated with Caribbean sponges Ircinia stroblina and Mycale laxissima
Source: Front Microbiol. 2014 Oct 28;5:561. doi: 10.3389/fmicb.2014.00561 (PMC4211547; doi:10.3389/fmicb.2014.00561)
Supplement: Supplementary file 1 [file DataSheet1.DOCX]

**Table S1 |** *nifH* gene OTUs found in sponge sample and their closest BLAST sequence matches.

| **Sponge- derived**  **90%-OTUs** | **No. of reads in each OTU per sponge species** | **Closest BLAST match**  **(accession no., %identity, source)** | **Closest cultivated microorganism**  **(accession no., %identity, source)** |
| --- | --- | --- | --- |
|  | **ISG ISC MLG MLC** |  |  |
| OTU01 | 673 3580 1310 4320 | EU594242.1 (100%)  Sponge RTMLH02 | KC256775.1 (88%)  *Leptolyngbya minuta* |
| OTU02 | 173 1967 989 2315 | HM601491.1 (92%)  Florida key reef water | AB264111.1 (84%)  *Cyanothece sp* |
| OTU03 | 338 2215 282 627 | EU594072.1 (96%)  Sponge IS15S | HQ906641.1 (99%)  *Mastigocladus testaurum* |
| OTU04 | 122 855 1688 3152 | KF657100.1 (88%)  Coral associated | FR669148.1 (84%)  *Klebsiella sp.* |
| OTU05 | 428 558 155 663 | GU594006.1 (95%)  Freshwater lake | DQ439648.1 (95%)  *Anabaena sphaerica* |
| OTU06 | 305 1458 198 1130 | EU594012.1 (93%)  Sponge IS3H07 | CP000781.1 (98%)  *Xanthobacter autotrophicus* |
| OTU07 | 977 978 804 0 | JF897530.1 (90%)  Microbial mats | CP005095.1(86%)  *Azotobacter vinelandii* |
| OTU08 | 0 0 195 1536 | AF414668.1 (85%)  Tropical sea grass | CP001614.2 (84%)  *Teredinibacter turnerae* |
| OTU09 | 627 0 0 0 | AF227929.1 (88%)  Marine stromatolite | KC992986.1 (85%)  *Okeania plumata* |
| OTU10 | 583 555 13 427 | KF657052.1 (96%)  Coral reef | GU238282.1 (95%)  *Hydrocoleum lyngbyaceum* |
| OTU11 | 426 1499 205 882 | KF657112.1 (90%)  Coral reef | FJ822999.1 (81%)  *Stenotrophomonas sp.* |
| OTU12 | 0 0 67 1179 | GU192789.1 (86%)  Intertidal Microbial mats | EU622788.1 (83%)  *Allochromatium minutissimum* |
| OTU13 | 573 1252 678 0 | DQ177014.1 (81%)  Mangrove roots | KF800047.1 (75%)  *Desulfobulbus alkaliphilus* |
| OTU14 | 578 485 212 0 | JQ514141.1 (89%)  Mountain river | AF013025.1 (90%)  *Microcoleus chthonoplastes* |
| OTU15 | 537 0 52 680 | HM601491.1 (87%)  Florida key seawater | EF397893.1(88%)  *Lyngbya wollei* |
| OTU16 | 474 668 8 0 | HQ634506.1 (94%)  Red Sea | KC256774.1 (85%)  *Hyella sp.* |
| OTU17 | 7 0 18 660 | DQ077995.1 (85%)  Marine sediment | KC256767.1 (85%)  *Leptolyngbya saxicola* |
| OTU18 | 225 794 192 0 | JN638712.1 (82%)  Black Sea seawater | CP001649.1(84%)  *Desulfovibrio salexigens* |
| OTU19 | 626 0 94 0 | KF854571.1 (87%)  Coral associated | AB189641.1 (86%)  *Halorhodospira halophila* |
| OTU20 | 101 0 98 0 | KF657141.1 (90%)  Coral associated | AJ297529.2(84%)  *Pseudomonas stutzeri* |
| OTU21 | 10 0 24 0 | EU594006.1 (99%)  Sponge IS3F08 | KC243670.1 (99%)  *Calothrix sp.* |
| OTU22 | 484 558 13 20 | AF216914.1 (90%)  Intertidal wetland | CDQ402935.1 (90%)  *Sulfitobacter sp.* |
| OTU23 | 23 433 88 0 | KF032146.1 (90%)  Soil | FJ347433.1(88%)  *Bradyrhizobium sp.* |
| OTU24 | 7 5 11 946 | KF846589.1 (87%)  Marine microbial mats | CP007053.1(85%)  *Opitutaceae bacterium* |
| OTU25 | 0 0 0 0 | KF854574.1 (81%)  Coral associated | CP001998.1(79%)  *Coraliomargarita akajimensis* |
| OTU26 | 0 0 0 0 | EU693410.1 (92%)  Coral associated | CP000143.2(88%)  *Rhodobacter sphaeroides* |
| OTU27 | 0 0 0 0 | AF216936.1 (82%)  Cordgrass associated | AB189641.1 (80%)  *Halorhodospira halophila* |

**FIGURE S1** | Community structure *I. strobilina*, *M. laxissima* and seawater based on 90% translated amino acid sequences similarity of *nifH* genes from gDNA and cDNA sources. (ISG1: gDNA from individual 1 of *I. strobilina*, ISG2: gDNA from individual 2 of *I. strobilina*, ISG3: gDNA from individual 3 of *I. strobilina*, ISC1: cDNA from individual 1 of *I. strobilina*, ISC2: cDNA from individual 2 of *I. strobilina*, ISC3: cDNA from individual 3 of *I. strobilina*, MLG1: gDNA from individual 1 of *M. laxissima*, MLG2: gDNA from individual 2 of *M. laxissima*, MLG3: gDNA from individual 3 of *M. laxissima*, MLC1: cDNA from individual 1 of *M. laxissima*, MLC2: cDNA from individual 2 of *M. laxissima*, MLC3: cDNA from individual 3 of *M. laxissima*).

**FIGURE S2** | Phylogenetic relationships of diazotrophic communities in sponges and water column based on *nifH* gene. Sequences from this study are highlighted in bold. Number in parentheses indicated the quantity of sequence reads from corresponding 454 pyrosequences. Tree topology constructed using maximum likelihood method with bootstrap values (>50%) indicated at the branch nodes. Archaeal *nifH* gene sequence from *Methanocaldococcus jannaschii* DSM 2661 (SAMN02603984) was used as outgroup.
