# Supplementary figures and images for: Temporal changes in the diazotrophic bacterial communities associated with Caribbean sponges Ircinia stroblina and Mycale laxissima
Source: Front Microbiol. 2014 Oct 28;5:561. doi: 10.3389/fmicb.2014.00561 (PMC4211547; doi:10.3389/fmicb.2014.00561)

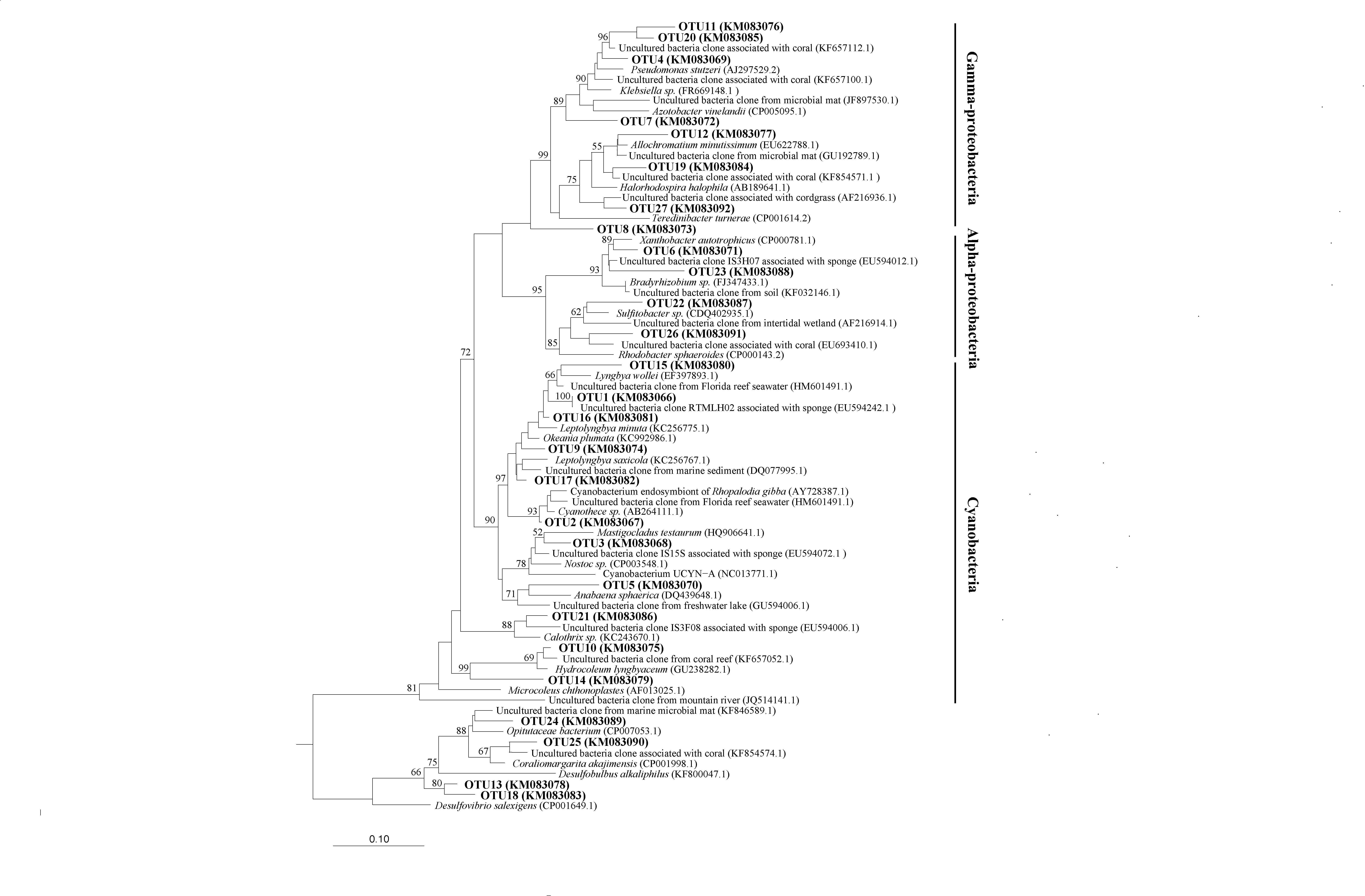

Supplement: Supplementary file 2 [file Image1.JPEG]

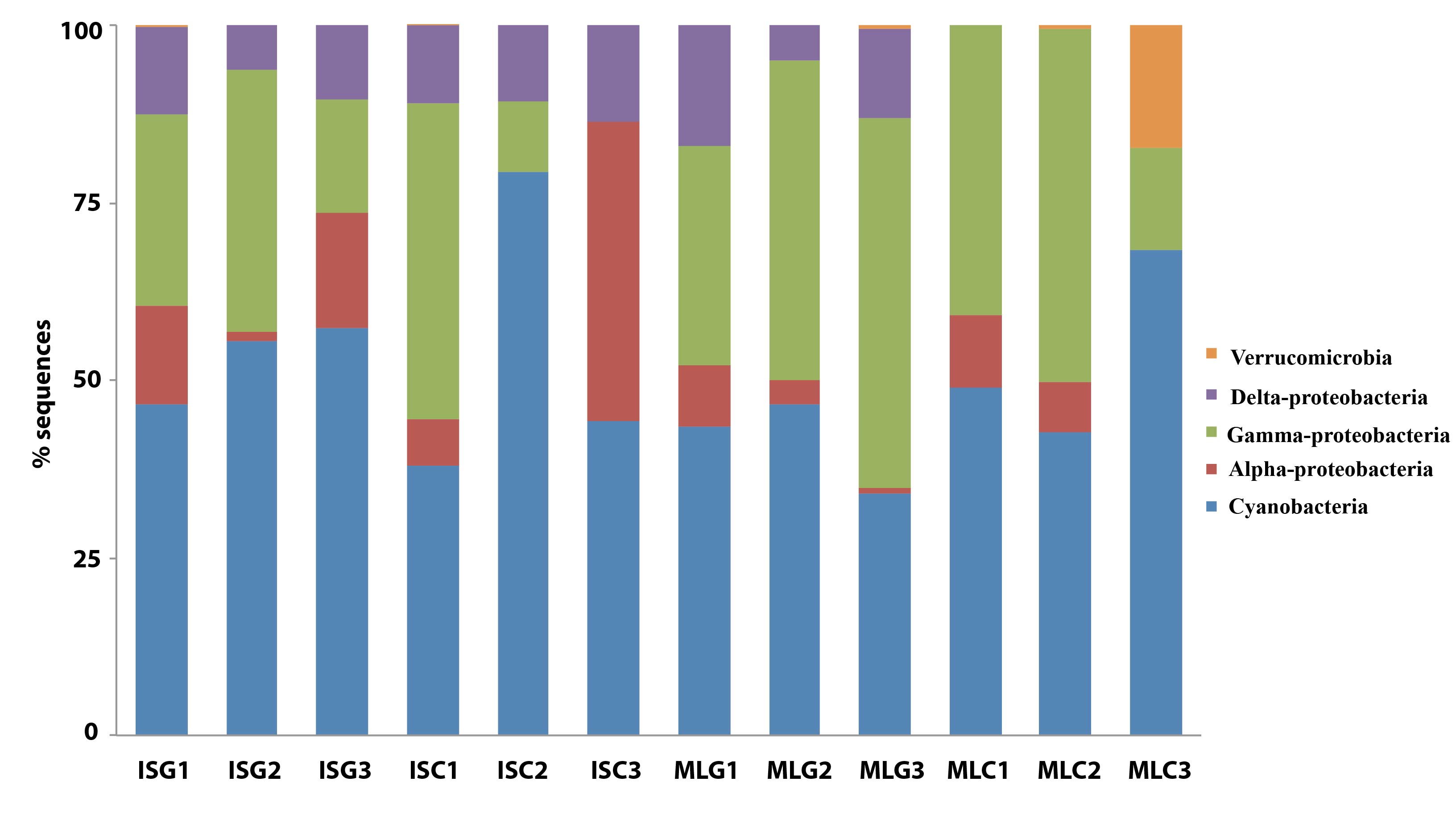

Supplement: Supplementary file 3 [file Image2.JPEG]
